# Genomic structural variation in Barramundi Perch *Lates calcarifer* and potential roles in speciation and adaptation

**DOI:** 10.1093/g3journal/jkae141

**Published:** 2024-06-27

**Authors:** Matthew A Campbell, Matthew C Hale

**Affiliations:** Centre for Carbon, Water and Food, The University of Sydney, 380 Werombi Road, Brownlow Hill, NSW 2570, Australia; Department of Biology, Texas Christian University, 2800 S. University Drive, Fort Worth, TX 76129, USA

**Keywords:** Asian sea bass, Barramundi, life history variation, partial migration, structural variation

## Abstract

Advancements in genome sequencing and assembly techniques have increased the documentation of structural variants in wild organisms. Of these variants, chromosomal inversions are especially prominent due to their large size and active recombination suppression between alternative homokaryotypes. This suppression enables the 2 forms of the inversion to be maintained and allows the preservation of locally adapted alleles. The Barramundi Perch (BP; *Lates calcarifer*) is a widespread species complex with 3 main genetic lineages located in the biogeographic regions of Australia and New Guinea (AUS + NG), Southeast Asia (SEA), and the Indian Subcontinent (IND). BP are typically considered to be a protandrous sequential hermaphrodite species that exhibits catadromy. Freshwater occupancy and intraspecific variation in life history (e.g. partially migratory populations) exist and provide opportunities for strongly divergent selection associated with, for example, salinity tolerance, swimming ability, and marine dispersal. Herein, we utilize genomic data generated from all 3 genetic lineages to identify and describe 3 polymorphic candidate chromosomal inversions. These candidate chromosomal inversions appear to be fixed for ancestral variants in the IND lineage and for inverted versions in the AUS + NG lineage and exhibit variation in all 3 inversions in the SEA lineage. BP have a diverse portfolio of life history options that includes migratory strategy as well as sexual system (i.e. hermaphroditism and gonochorism). We propose that the some of the life history variabilities observed in BP may be linked to inversions and, in doing so, we present genetic data that might be useful in enhancing aquaculture production and population management.

## Teaser

Barramundi Perch, aka Asian Sea Bass or ‘Barramundi’, are widely distributed in the tropical Indo-Pacific and are a popular recreational and important aquaculture fish. Molecular data has identified three divergent genetic lineages and indicate that Barramundi Perch is better described as a species complex. Campbell and Hale identify genetic architecture that may underpin the speciation and adaptation of this species complex across its broad range. Their results may be leveraged for evolutionary study, aquaculture development, management, and conservation.

## Introduction

The decreased cost of whole-genome sequencing, coupled with the increased power of modern-day computational analyses, has made it easier to generate genetic maps and find structural variants within the genomes of nonmodel organisms (e.g. Flagel *et al*. 2019). The most frequently described structural variant is chromosomal inversions (hereafter referred to as inversions) due to their large size—often in the order of several megabases—that allows for their identification and the determination of different homokaryotypes across the range of the species in question. Interest in inversions stems largely from their ability to suppress recombination between different homokaryotypes, which allows for the maintenance of locally adapted alleles (Kirkpatrick 2010; Schwander *et al*. 2014; Wellenreuther and Bernatchez 2018; reviewed in Huang *et al*. 2020). Within fishes, a key example is the association between the ∼55 Mb Omy05 inversion in rainbow trout (*Oncorhynchus mykiss*) and migratory behavior (i.e. residency vs anadromy) as well as other phenotypes such as phototransduction, development rate, and sexual maturity (Nichols *et al*. 2007; Miller *et al*. 2012; Pearse *et al*. 2014, 2019). Furthermore, some of the key genes associated with life history development in Omy05 have been found in inversions in nonsalmonids, suggesting not only an emerging paradigm of inversions in fishes, but also a conservation in the genes contained in those inversions (MacGuigan *et al*. 2023).

**Fig. 1.**
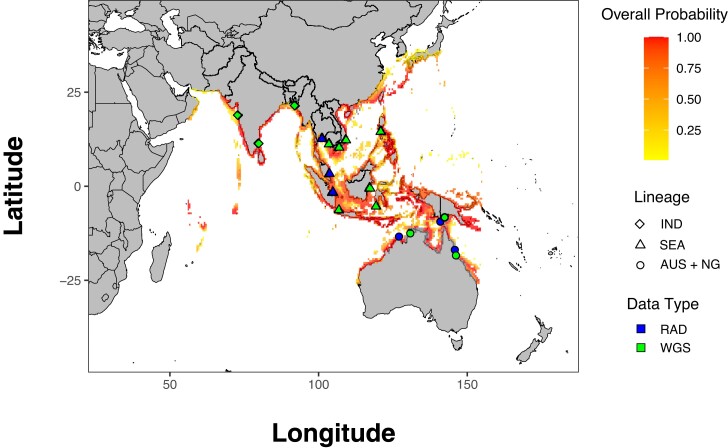
A range map of *L. calcarifer* based on the probability of occurrence, “overall probability” (Kaschner *et al*. 2015) with sampling locations included in this study. The lineage of fish sensu Campbell and Becker (under review) is indicated as well as data type.

The Barramundi Perch (BP; *Lates calcarifer*) is a wide-ranging protandrous hermaphroditic species (50° E to 160° W of longitude, and from 24° N to 25° S of latitude; e.g. FAO 2009; Mathew 2009; Fig. 1). The life history of BP varies, but most studies find that the species exhibits catadromy, with mature fish spawning in saline environments and subsequent migration of juveniles into freshwater as males. After maturation, they return to saline environments to spawn and remain for the rest of their life cycle where males transition to females after a period of several years (e.g. Moore 1979; Moore and Reynold 1982; Balston 2009). The fast growth, high fecundity, ability to be cultured, and wide environmental tolerance of BP have led to its recent rapid expansion in aquaculture (FAO 2009). Importance in aquaculture has led to a concomitant increase in genetic studies of BP, yielding a high-quality reference genome and genome-wide sequence data from across the species range (Vij *et al*. 2016; Wang *et al*. 2016).

The genetic data identify deep divergences within BP corresponding to 3 main lineages, 1 located around the Indian subcontinent and the Bay of Bengal (IND), a second in Southeast Asia (SEA), and a third in Australia and New Guinea (AUS + NG; Ward *et al*. 2008; Vij *et al*. 2014; Campbell and Becker under review). The IND lineage appears to be the most distinct, splitting from a combined SEA and AUS + NG lineage ∼7 MYA, with separation of the SEA and AUS + NG lineages occurring ∼1 MYA (Campbell and Becker under review). There appears to be a genetic substructure within both the IND lineage and the AUS + NG lineage (e.g. Shaklee and Salini 1985; Loughnan *et al*. 2019; Campbell and Becker under review), whereas the SEA lineage has large parts of the range in its western distribution (e.g. western Malaysia, western Indonesia, and Thailand) without a genetic structure, and the other parts of its range show a high degree of genetic differentiation (e.g. Sulawesi and the Philippines; Wang *et al*. 2016; Campbell and Becker under review). Also, there appears to be introgression between the IND and the SEA lineages that likely represent either a natural hybrid zone or a result of translocations—and subsequent escape or mixing in hatcheries—from aquaculture (e.g. around Thailand: Yue *et al*. 2009; Vij *et al*. 2014, 2016; Campbell and Becker under review).

BP exhibit a diverse portfolio of life histories and divergent selection pressures across the species’ range. For example, AUS + NG lineage BP appear to undergo limited seaward migrations and exhibit a high degree of genetic structuring. The effects of gene flow are reduced in AUS + NG lineage BP, making the development of an inversion less likely than species with active introgression (e.g. Shaklee and Salini 1985; Marshall 2005; reviewed in Wellenreuther and Bernatchez 2018; Huang *et al*. 2020). Studies on life history development and variation outside Australia and New Guinea are few (Parazo *et al*. 1998), but genetic data indicate (1) a lack of a population genetic structure suggestive of dispersal through marine habitats and gene flow, and (2) hybridization in Southeast Asia between SEA and IND lineages (Campbell and Becker under review). Both conditions are conducive to the creation of inversions, as selection favors mechanisms that preserve locally adaptive alleles.

To date, the increasing number of genomic resources for BP has not been thoroughly investigated for the presence and distribution of structural variants. Therefore, we leveraged preexisting genomic data to document and describe novel inversions across the range of BP. We characterize the phylogenetic and geographic distribution variations of these inversions using RADseq and WGS datasets and test for a homology of candidate inversions with the well-documented Omy05 inversion in rainbow trout. We provide a hypothesis for the phenotypes arising from candidate inversions and a potential role in speciation with gene flow. Structural variation within BP merits additional study and has numerous practical implications for the conservation and management of this important taxon as well as advancing the study of the genomic basis for speciation.

## Methods

### Sequence data and genotype calling

We obtained genome-wide sequence data from a total of 190 BP from across the range of this species, specifically, from India and Bangladesh (13 samples), Southeast Asia (106 samples), and Australasia (71 samples: see Table 1 for more details on the samples used in this study). Two data types from the NCBI Sequence Read Archive were used, whole-genome sequence data (WGS: 60 samples from all 3 areas, BioProject accession numbers PRJNA311498 and PRJNA1021005) and RADseq data (RAD: 130 samples from Southeast Asian and Australasian areas, BioProject accession PRJDB3890). All data were aligned to the BP reference genome GCF001640805.2 (Vij *et al*. 2016) using the Burrows–Wheeler Aligner v. 0.7.17, specifying the mem algorithm with default parameters (Li and Durbin 2009, 2010). Alignments were then sorted, filtered for proper read pairs, and PCR duplicates removed using SAMtools v. 1.19 (Li *et al*. 2009; Danecek *et al*. 2021). Metrics of read count, filtered read count, and depth of coverage were computed with SAMtools. Samples with ≤3× coverage were excluded from further analyses.

**Table 1.** Samples examined in this study, which include WGS samples previously analyzed by Vij *et al*. (2016)^1^ and Campbell and Becker (under review) and RAD sequencing samples reported by Wang *et al*. (2016)^2^.

Region	Source	Sample size	Lineage	Data type	BioProject	Source publication
Northern Territory, Australia	Wild-Caught	6	AUS + NG	WGS	PRJNA311498	1
Queensland, Australia	Wild-Caught	6	AUS + NG	WGS	PRJNA311498	1
Papua New Guinea	Wild-Caught	5	AUS + NG	WGS	PRJNA311498	1
Indonesia	Unknown	5	SEA	WGS	PRJNA311498	1
Indonesia	Wild-Caught	5	SEA	WGS	PRJNA311498	1
Indonesia	Unknown	1	SEA	WGS	PRJNA311498	1
Philippines	Hatchery Broodstock	5	SEA	WGS	PRJNA311498	1
Vietnam	Hatchery Broodstock	2	SEA	WGS	PRJNA311498	1
Cambodia	Wild-Caught	5	SEA	WGS	PRJNA311498	1
Thailand	Wild-Caught	7	SEA	WGS	PRJNA311498	1
Bangladesh	Unknown	2	IND	WGS	PRJNA1021005	
India Eastern Coast	Wild-Caught	7	IND	WGS	PRJNA311498	1
India Western Coast	Wild-Caught	4	IND	WGS	PRJNA311498	1
Australia East “AUE”		14	AUS + NG	RAD	PRJDB3890	2
Australia West “AUW”		22	AUS + NG	RAD	PRJDB3890	2
Papua New Guinea “PNG”		18	AUS + NG	RAD	PRJDB3890	2
Indonesia “INA”		24	SEA	RAD	PRJDB3890	2
Malaysia “MAL”		23	SEA	RAD	PRJDB3890	2
Thailand “THA”		29	SEA	RAD	PRJDB3890	2

The region of origin, genetic lineage, and BioProject IDs are included. BioProject PRJNA311498 is 100 bp paired-end data generated by an Illumina HiSeq2500, BioProject PRJNA1021005 is 150 bp paired-end data generated by an MGI Tech DNBSEQ-G400, and BioProject PRJDB3890 data are 150 bp paired-end sequencing generated on an Illumina NextSeq500.

Genotypes were called from the combined dataset of both WGS and RAD with Analysis of Next Generation Sequence Data (ANGSD v. 0.93) from the 24 chromosomes of the BP reference genome (Korneliussen *et al*. 2014). In order to ensure that any candidate SNP was not caused by differences in the sequencing method (i.e. WGS vs RAD), we used stringent parameters in ANGSD, i.e. present in ≥90% of individuals, a minimum minor allele frequency of 0.05, a minimum base quality of 20, a minimum mapping quality of 20, a posterior cutoff value of 0.90, and a *P-*value of 1 × 10^−6^. A SAMtools genotype likelihood model was specified and the output files written in both geno and PLINK formats. The PLINK-formatted file was converted to a VCF file with PLINK v. 1.90 (Purcell *et al*. 2007). A “pruned” version was created by removing linked SNPs with BCFTools + prune (-l 0.20 -w 10000). These steps were repeated with only the RAD data and only the WGS data separately.

### Genome-wide population genetic signal

We examined the overall signal in combined dataset of WGS and RAD data with a principal component (PC) analysis to verify the separation of samples into 3 main lineages (AUS + NG, SEA, and IND) and to also verify that data type (WGS vs RAD) was not a main contributor to variation within the combined dataset. The combined pruned data were imported into R (R Development Core Team 2024) and a PC analysis conducted with snpR (Hemstrom and Jones 2023) and visualized with ggplot2 (Wickham 2009). Individual heterozygosity was calculated genome wide with the combined pruned dataset with the calc_hs() function of snpR (Hemstrom and Jones 2023) and visualized as boxplots with ggplot2 (Wickham 2009).

### Identification and distribution of potential inversions

We searched for potential inversions by applying a local PC analysis, examining linkage disequilibrium (LD), and examining patterns of heterozygosity and F_ST_ between alternative karyotypes of candidate inversions (e.g. Huang *et al*. 2020; Hale *et al*. 2021). A local PC analysis identifies regions of the genome with a population genetic structure that differs from the majority of the genome and is implemented in the R package lostruct (Li and Ralph 2019). We used the unpruned called SNP dataset of combined RAD and WGS data and split it into 1 Binary variant Call Format (BCF) file with BCFTools per chromosome (Danecek *et al*. 2021). We then ran the “run_lostruct.R” R script from the lostruct package to conduct the local PC analysis using nonsliding windows of 50 SNPs and retaining the first 3 multidimensional scaling (MDS) axes. We then identified outlier windows along each MDS axis with the boxplot.stats function of R (for more details, see Hale *et al*. 2021).

Linkage disequilibrium was calculated across chromosomes with identified outliers from the results of lostruct analysis (details above) using PLINK v1.9 (Purcell *et al*. 2007). We calculated *R*^2^ on a per chromosome basis with both the combined pruned dataset and the unpruned RAD dataset with PLINK (--r2 inter-chr –ld-window-r2 0.3 –allow-extra-chrom –double-id). We visualized LD patterns with ggplot2 filtering for *R*^2^ values >0.8 to help identify regions of the genome with extended high LD.

Patterns of heterozygosity were assessed with a PC analysis of candidate inversion zones to test for 3 main clusters of ancestral homokaryotypes, heterozygotes, and inverted homokaryotypes. Candidate diagnostic SNPs were identified by selecting SNPs with the highest 5% of loadings contributing to the first PC and used to determine the inversion genotype of individuals. Individual heterozygosity was calculated from the candidate inversions with the calc_hs() function of snpR (Hemstrom and Jones 2023) and boxplots generated with ggplot2 (Wickham 2009) of ancestral homokaryotypes (AHom), heterozygotes (Het), and inverted homokaryotypes (Rhom). Frequencies of inversion genotypes for each sampling location were plotted geographically with ggplot2 (Wickham 2009).

We calculated *F*_ST_ between alternative homokaryotypes in the SEA lineage (due to lower sample sizes in the IND and AUS lineages) to further verify boundaries and the presence of inversions. The RAD dataset was filtered to only homokaryotypes from the SEA lineage, then a case–control design in PLINK was used to calculate *F*_ST_. Ancestral homozygotes were designated as “1” and inverted homozygotes as “2.”

### Homology ascertainment for rainbow trout

The MCscan pipeline (Tang *et al*. 2008) was used to search for homologous blocks in the BP genome and the rainbow trout genome (GCF 013265735.2). Due to genomic redundancy as a result of a salmonid-specific whole-genome duplication leading to similar genetic backgrounds having similar functions in rainbow trout (Campbell *et al*. 2021), we also compared the BP genome with the northern pike (*Esox lucius*) genome (GCF_011004845.1). Pikes and their relatives did not undergo a fourth genome duplication and are the sister lineage to the salmonids, and northern pike can serve as an ancestral “protokaryotype” for comparison with salmonids (e.g. López *et al*. 2004; Campbell *et al*. 2013; Blumstein *et al*. 2020). A pairwise synteny search was conducted that utilized protein-coding genes as part of the MCscan pipeline and macrosynteny visualized between the rainbow trout Omy05 and Omy20 chromosomes known to contain large chromosomal inversions, the northern pike genome, and the BP genome.

### Functional characterization

We used a blast approach to determine the functions of protein-coding genes found within the candidate inversions. Briefly, protein-coding sequences for all 25,072 genes within the BP genome were downloaded and annotated against the UniProt reference protein database using BLASTX with default parameters (apart from: maximum *e*-value = 1.0*e*−10, maximum number of blast hits saved per sequence = 15). Blast hits were then uploaded into Blast2GO v6 (Conesa *et al*. 2005) to obtain GO terms and GO-SLIM terms associated with the protein sequences. The generic GO-SLIM database was used as available at geneontology.github. Fisher's exact tests were used to test for enrichment of GO and GO-SLIM terms associated with protein-coding genes within each candidate inversions and the rest of the protein-coding genes within the BP genome. Significantly enriched GO and GO-SLIM terms were identified using a Benjamini–Hochberg False Discovery Rate (FDR)-corrected *P*-value (alpha = 0.05) and that the GO term had to be present in at least 10 different protein-coding genes within the inversion.

## Results

### Sequence data, genotype calling, and genome-wide signal

**Fig. 2.**
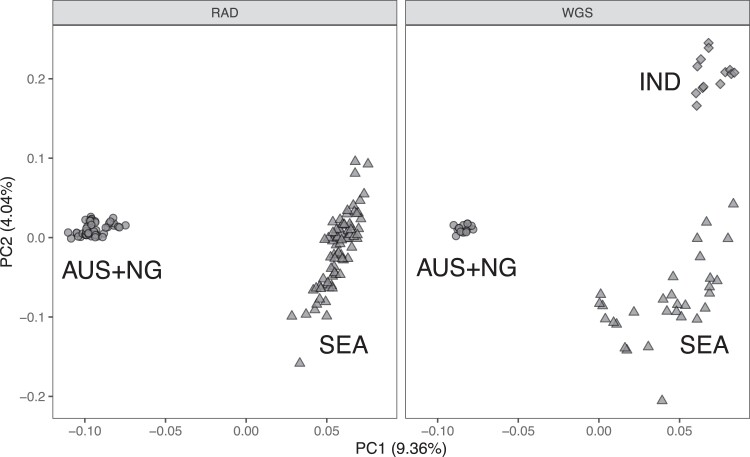
A principal component analysis of combined RAD and WGS datasets from 16,306 called SNPs present after pruning linked SNPs. The first 2 PCs are shown with the plot facetted between RAD (*n* = 130) and WGS (*n* = 60) data types. The main lineage AUS + NG (*n* = 71), SEA (*n* = 106), and IND (*n* = 13) are indicated by shape.

A total of 60 WGS samples from 13 locations and 130 RAD samples from 6 localities were analyzed (Fig. 1, Table 1). The combined dataset produced 104,440 called genotypes that after pruning numbered 16,306 SNPs. Genome-wide PC analysis of the combined dataset is presented as Fig. 2, indicating 3 clear genetic clusters comprising the AUS + NG (*n* = 71), SEA (*n* = 106), and IND (*n* = 13) lineages. The first PC axis (PC1) exhibits 9.36% of variance and separates the AUS + NG lineage from all other samples. The second PC axis (PC2, 4.04% of variance), corresponds to variation within the SEA lineage as well as separating the IND lineage from all other samples. Median individual heterozygosity is highest in the SEA lineage with samples sequenced by both RAD and WGS methods, with AUS + NG and IND lineage fish roughly equivalent (Supplementary Fig. 1).

### Identification and distribution of potential inversions

**Fig. 3.**
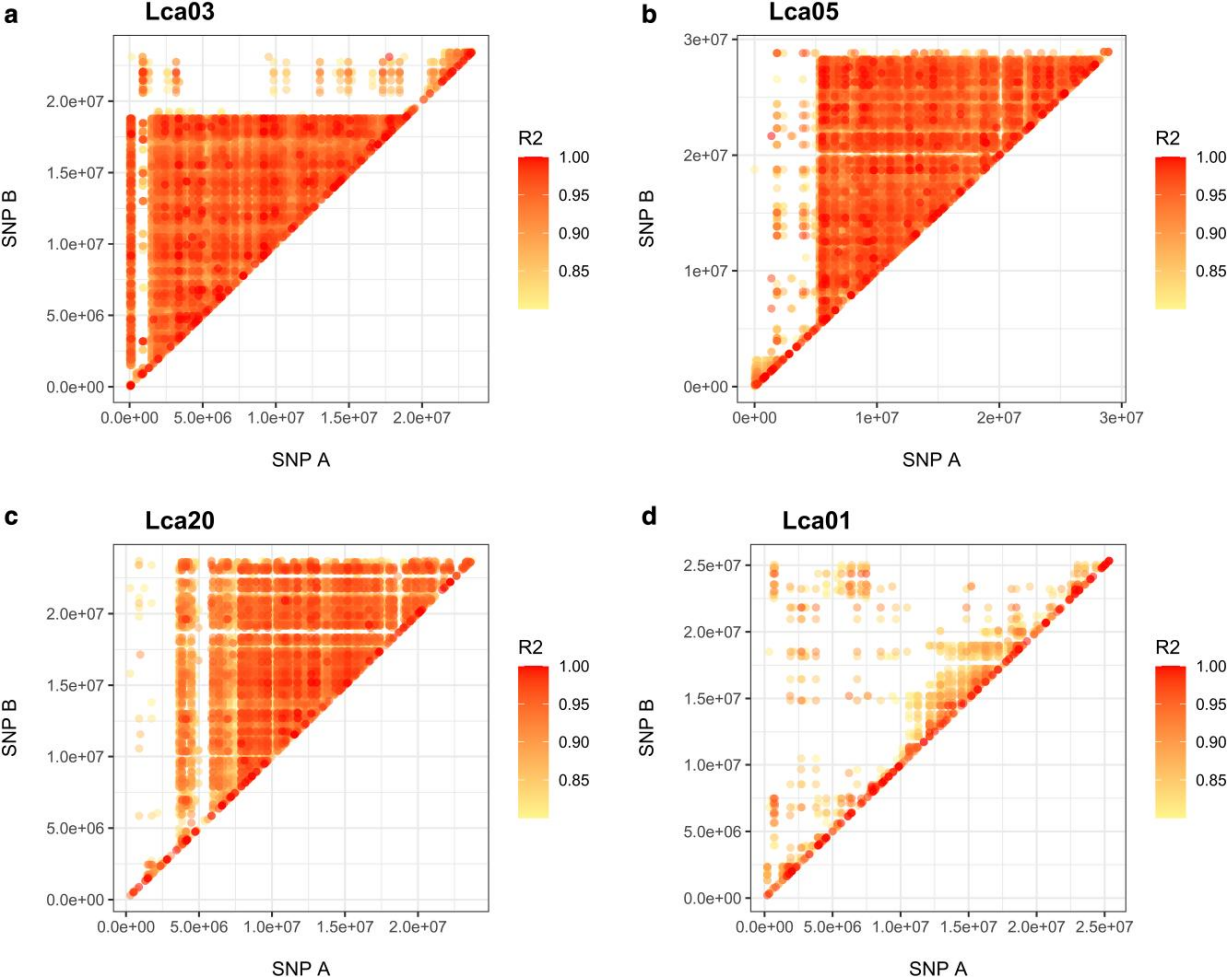
A linkage disequilibrium analysis of a combined dataset (16,306 SNPs genome wide after pruning) of 3 chromosomes exhibiting increased LD [(a) Lca03, (b) Lca05, (c) Lca20] and a fourth chromosome without increased LD for comparison [(d) Lca01]. The *x-*axis is the position of an SNP, “SNP A,” with the *y-*axis being the position of a second SNP, “SNP B.” Each point is color-coded to the measurement of LD (R2).

**Fig. 4.**
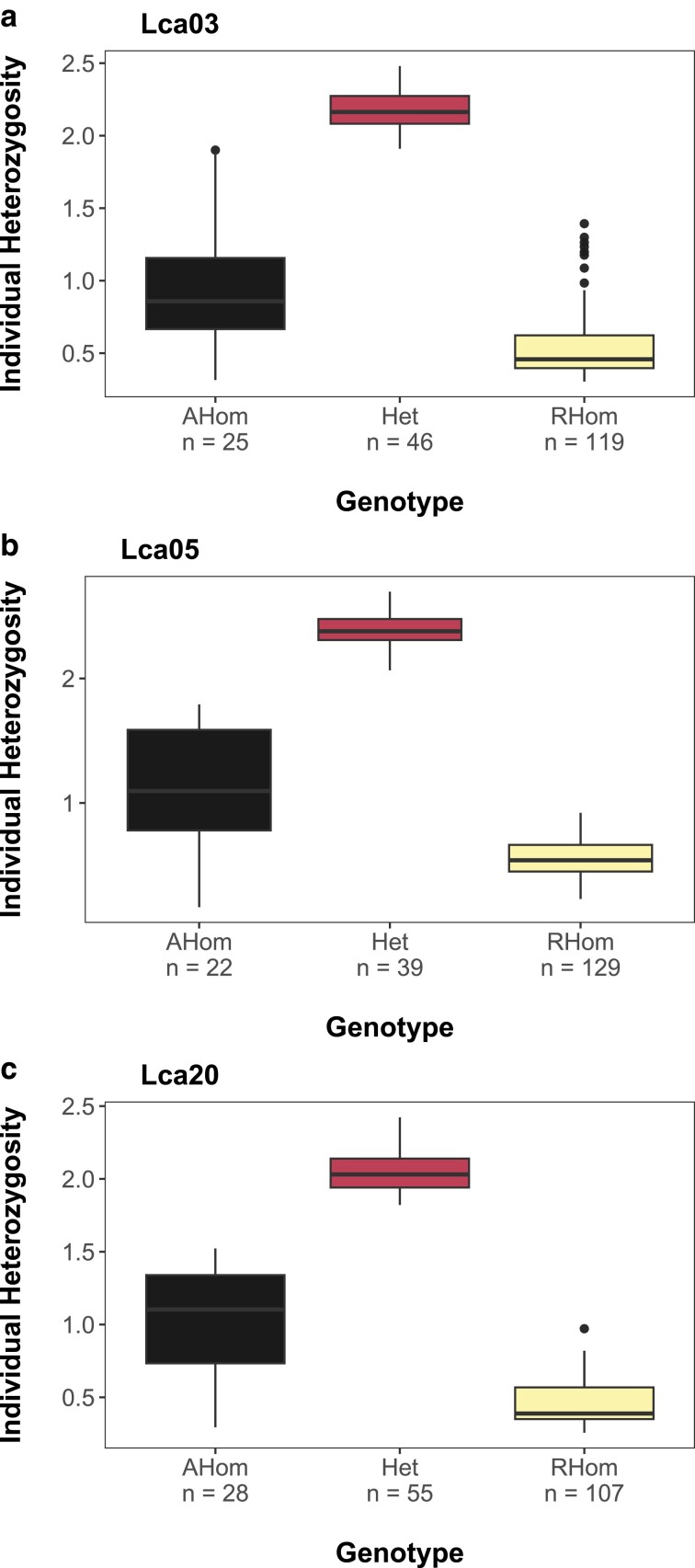
Individual heterozygosity boxplots for 3 candidate chromosomal inversions (inversions) on (a) Lca03, (b) Lca05, and (c) Lca20. The number of individuals of each genotype is included on the *x—*axis.

The first MDS axis from local PC analysis has few outliers (*n* = 25) with the highest proportion a located on Lca05, 36.0% (9/25). The next chromosome with a substantial number of outliers on MDS1 is Lca03 with 20.0% (5/25). The remaining outliers were widely dispersed across the BP genome. Of the 86 outliers on MDS2, 48.8% (42/86) are located on Lca03 and 41.9% (36/86) on Lca05 (Supplementary Fig. 2). Similarly, MDS3 outliers were clustered on Lca20 (40.5%, 47/116), Lca03 (∼35%), and Lca05 (∼15%), indicating variation in these regions of the genome reflects a different population structure than most of the genome. LD calculated from 16,306 SNPs in the pruned combined dataset and the 76,601 SNPs in the unpruned RAD dataset reveals strong LD across large regions of Lca03 (∼17 Mb), Lca05 (∼23 Mb), and Lca20 (∼20 Mb; Fig. 3, Supplementary Fig. 3), also suggesting candidate inversions. PC analysis of regions of high LD on the 3 BP chromosomes mentioned above suggests a splitting of 3 clusters along PC1 with the separation of candidate inverted homozygotes of the AUS + NG lineage from inverted homozygotes of the SEA lineage on PC2 (Supplementary Fig. 4). Patterns of heterozygosity indicate moderate amounts of variation in putative ancestral homokaryotypes, elevated amounts in heterozygotes, and reduced amounts in putative inverted homokaryotypes for all 3 candidate inversions (Fig. 4).

**Fig. 5.**
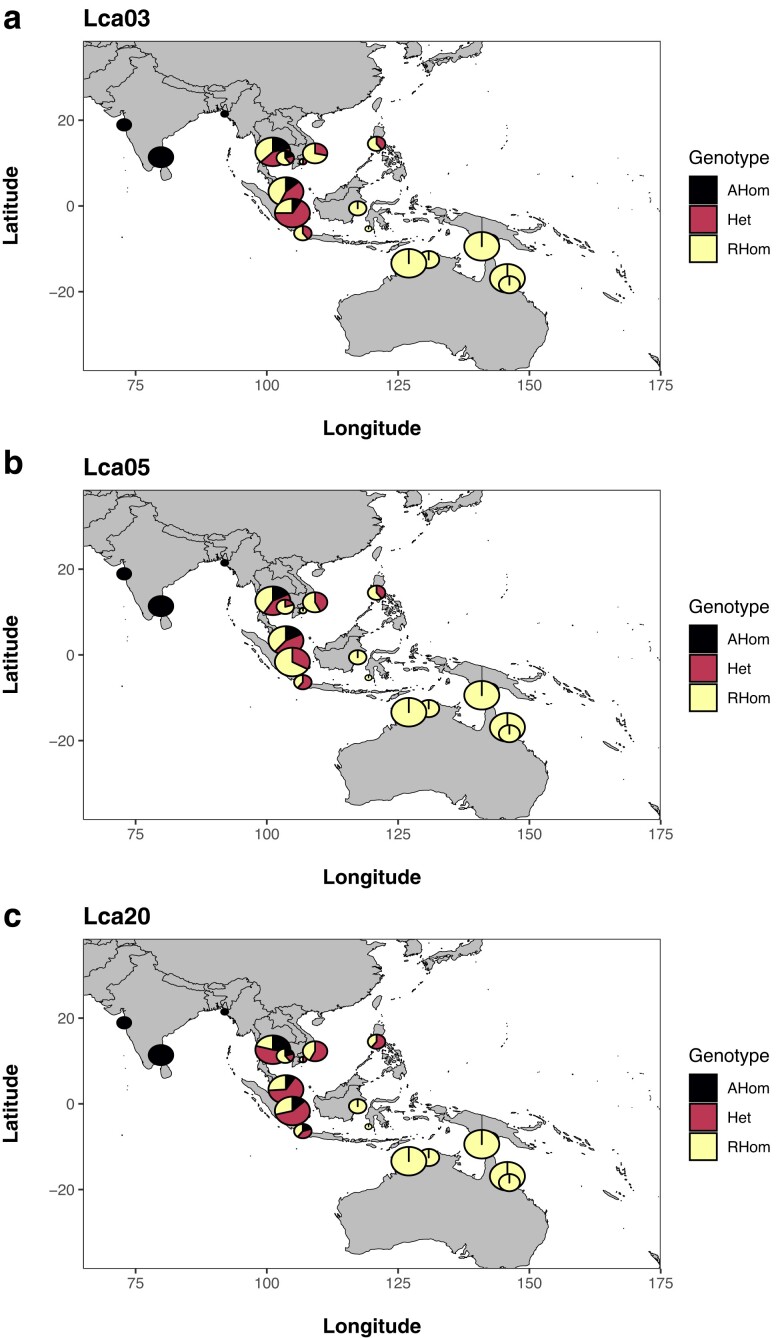
A geographic distribution of candidate chromosomal inversion (inversion) genotypes from (a) Lca03, (b) Lca05, and (c) Lca20. The size of individual pie charts is scaled to reflect sample sizes.

**Fig. 6.**
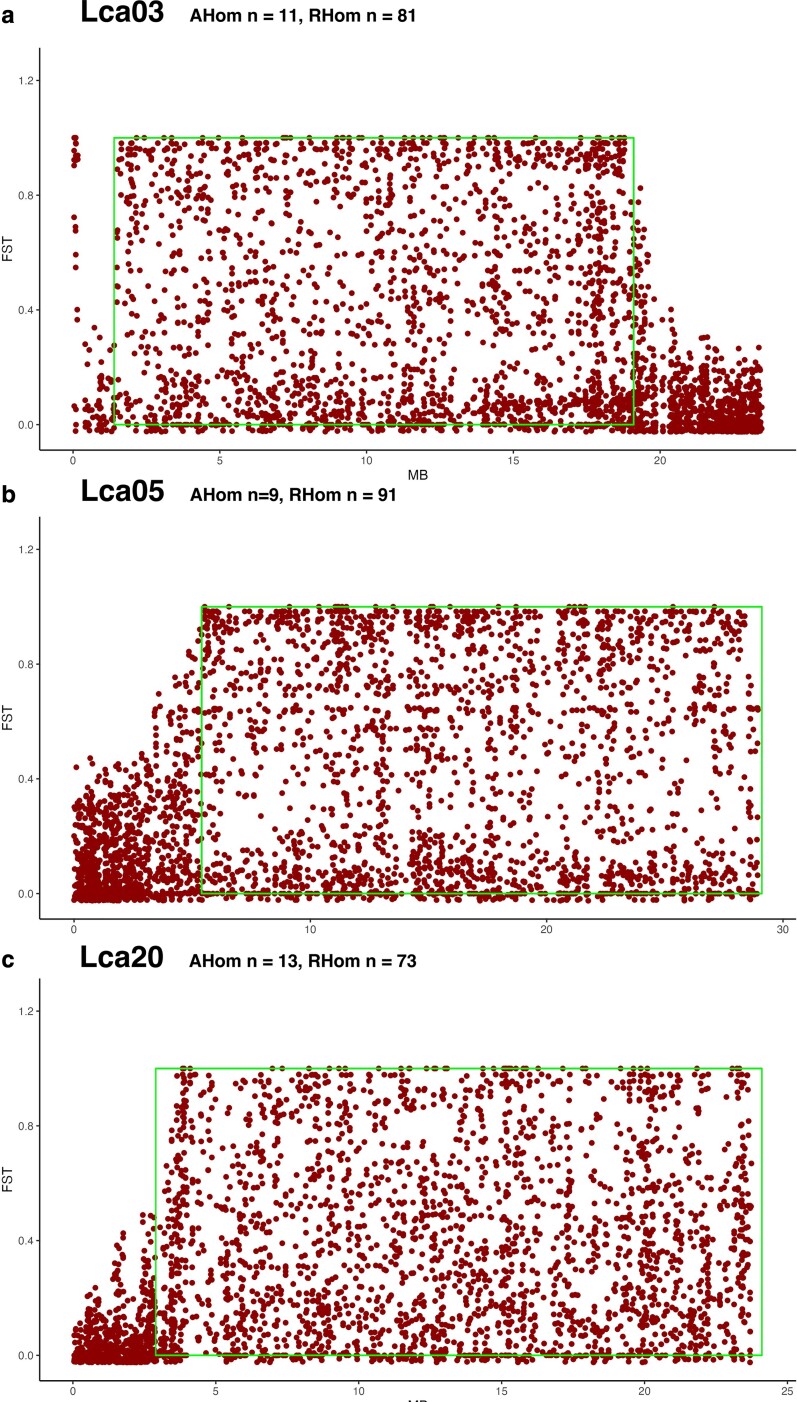
*F*_ST_ calculations for 3 candidate inversions [(a) Lca03, (b) Lca05, (c) Lca20]. *F*_ST_ values were calculated between putative homokaryotypes from the Southeast Asian genetic lineage of the RAD dataset. The approximate boundaries of the inversions are outlined. Sample sizes are provided in the figure.

Distribution of all 3 inversions follows an East to West gradient (Fig. 5, Supplementary Fig. 5), and we report candidate inversion genotypes for each sample analyzed in Supplementary Table 1. Ancestral homokaryotypes are dominant in the IND lineage from 3 sampling locations (India Western Coast, India Eastern Coast, and Bangladesh, total *n* = 13), with a mixture of genotypes found in sampling locations in Thailand, Peninsular Malaysia, the islands of Sumatra and Java, Vietnam, Cambodia, and the Philippines. The islands of Borneo, Sulawesi, New Guinea, and Australian sampling locations contained only homozygotes for the inverted homokaryotype. *F*_ST_ scans confirmed the identification of all 3 candidate inversions as shown by increases in *F*_ST_ values between homokaryotypes (Fig. 6).

### Homology ascertainment to rainbow trout

**Fig. 7.**
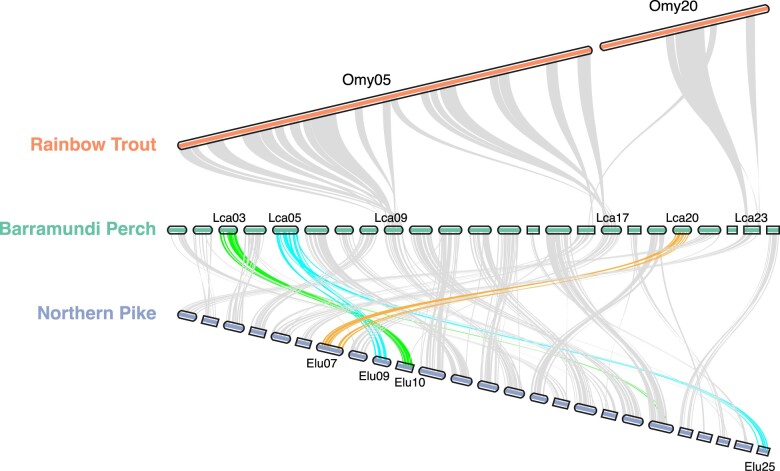
A macrosyntenic comparison of rainbow trout Omy05 and Omy20 chromosomes with the Barramundi Perch (BP) genome, and the BP genome with the northern pike genome.

Macrosyntenic comparison identified a high degree of homology between Omy05 and Lca09 and Omy20 and Lca23, not the candidate inversion containing chromosomes of Lca03, Lca05, or Lca20 (Fig. 7). Alignment of BP to the northern pike genome indicated homology between Lca03 and Elu10, Lca05 and Elu09/Elu25, and Lca20 and Elu07.

### Functional characterization

A total of 1771 protein-coding genes were found within the 3 candidate inversions of which 530 were in the inversion on Lca03, 492 were within the inversion on Lca05, and 749 were within the inversion on Lca20. Fisher's exact tests comparing GO terms associated with the protein-coding genes within each candidate inversion against all other protein-coding genes found 5 GO terms enriched for genes within the inversion on Lca03, none enriched for genes within the inversion on Lca05, and 84 terms enriched for genes within the inversion on Lca20. Of those enriched terms, all were overrepresented, and 1 was within the biological process category for Lca03 and 36 for Lca20 (see Table 2 for a list of enriched biological process GO terms). GO-SLIM enrichment analyses found 8 terms overrepresented in the Lca03 inversion, and 13 terms overrepresented in the Lca20 inversion. Investigating the functions of the enriched biological processes showed many terms associated with development, and ionic balance all of which might be important in the development of catadromy. GO-SLIM enrichment strongly suggests overexpression of proteins involved in DNA and cell binding in the Lca03 inversion and cell transport in the Lca20 inversion (see Table 3 for a list of enriched GO-SLIM terms). However, it is important to note that there were a lot of variations in the terms associated with the Lca20 inversion, suggesting the role of the genes is many and varied.

**Table 2.** Enriched GO terms associated with protein-coding genes located in the Lca03 (above the line) and Lca20 (below the line) candidate inversions.

GO ID	GO term (biological processes)	FDR-corrected *P*-value
GO:0044271	Cellular nitrogen compound biosynthetic process	0.03
GO:0060731	Positive regulation of intestinal epithelial structure maintenance	0.004
GO:0070715	Sodium-dependent organic cation transport	0.004
GO:0051179	Localization	0.004
GO:0042391	Regulation of membrane potential	0.005
GO:0051234	Establishment of localization	0.005
GO:0099505	Regulation of presynaptic membrane potential	0.005
GO:0016338	Calcium-independent cell–cell adhesion via plasma-membrane cell-adhesion molecules	0.007
GO:0015879	Carnitine transport	0.008
GO:0001554	Luteolysis	0.008
GO:0060456	Positive regulation of digestive system process	0.008
GO:1902270	(R)-carnitine transmembrane transport	0.011
GO:0060078	Regulation of postsynaptic membrane potential	0.011
GO:0055085	Transmembrane transport	0.011
GO:0150111	Regulation of transepithelial transport	0.011
GO:1903348	Positive regulation of bicellular tight junction assembly	0.011
GO:0014045	Establishment of endothelial blood-brain barrier	0.012
GO:1900749	(R)-carnitine transport	0.012
GO:0009609	Response to symbiotic bacterium	0.012
GO:0050772	Positive regulation of axonogenesis	0.012
GO:0034330	Cell junction organization	0.013
GO:0006810	Transport	0.014
GO:0050925	Negative regulation of negative chemotaxis	0.015
GO:0060730	Regulation of intestinal epithelial structure maintenance	0.015
GO:0098742	Cell–cell adhesion via plasma-membrane adhesion molecules	0.016
GO:0006811	Monoatomic ion transport	0.020
GO:1905048	Regulation of metallopeptidase activity	0.022
GO:0007156	Homophilic cell adhesion via plasma-membrane adhesion molecules	0.022
GO:0009437	Carnitine metabolic process	0.025
GO:0099565	Chemical synaptic transmission, postsynaptic	0.025
GO:1902603	Carnitine transmembrane transport	0.027
GO:1904862	Inhibitory synapse assembly	0.030
GO:0071420	Cellular response to histamine	0.030
GO:0006996	Organelle organization	0.037
GO:0051129	Negative regulation of cellular component organization	0.040
GO:0034220	Monoatomic ion transmembrane transport	0.046
GO:0034329	Cell junction assembly	0.047

Only biological process GO terms are shown. All enriched GO terms were required to be observed in at least 10 different protein-coding genes within the candidate inversions.

**Table 3.** Enriched GO-SLIM terms associated with protein-coding genes within candidate inversions compared with protein-coding genes in other regions of the genome.

GO ID	GO-SLIM term	Category	FDR-corrected *P*-value
GO:0003676	Nucleic acid binding	MF	0.0036
GO:0005654	Nucleoplasm	CC	0.0036
GO:0070013	Intracellular organelle lumen	CC	0.0036
GO:0031981	Nuclear lumen	CC	0.0036
GO:0031974	Membrane-enclosed lumen	CC	0.0036
GO:0043233	Organelle lumen	CC	0.0036
GO:0097159	Organic cyclic compound binding	MF	0.0036
GO:0003677	DNA binding	MF	0.0145
GO:0005215	Transporter activity	MF	0.0019
GO:0051179	Localization	BP	0.0019
GO:0055085	Transmembrane transport	BP	0.0028
GO:0034330	Cell junction organization	BP	0.0030
GO:0006810	Transport	BP	0.0030
GO:0051234	Establishment of localization	BP	0.0031
GO:0110165	Cellular anatomical entity	CC	0.0042
GO:0016020	Membrane	CC	0.0228
GO:0005783	Endoplasmic reticulum	CC	0.0249
GO:0016043	Cellular component organization	BP	0.0249
GO:0009987	Cellular process	BP	0.0249
GO:0007010	Cytoskeleton organization	BP	0.0290
GO:0005886	Plasma membrane	CC	0.0423

GO-SLIM terms enriched in the Lca03 inversion are listed above the line and Lca20 below the line. GO categories are split into cellular component (CC), molecular function (MF), and biological process (BP).

## Discussion

Here, we re-analyzed previously collected genomic data from across the range of BP to identify and document novel chromosome inversions. Utilizing genome sequencing data and sampling from across the species range is important for documenting novel inversions as our analyses showed that different populations of BP are fixed for different forms of all 3 inversions. For example, western populations (Fig. 5) show fixation of the ancestral form of all 3 candidate inversions in the IND lineage BP (Supplementary Fig. 5). Moving East, there is a zone with high amounts of variation in the 3 candidate inversions in a range of ∼100–110° E longitude (Fig. 5) and is composed of fish from the SEA lineage that exhibit little genetic structuring and potential hybridization with the IND lineage (Campbell and Becker under review). The AUS + NG lineage lacks variation in the candidate inversions and is fixed for all 3 inverted forms (Supplementary Fig. 5). Investigation into the literature corroborates these findings, with a karyotype of 19 telocentric chromosomes, 1 subtelocentric, 3 submetacentric, and 1 metacentric identified from Indian BP exhibiting no variation (Khuda-Bukhsh 1979; Sudesh and John 1993). On the contrary, Australian BP differ in having 3 subtelocentric chromosomes and 1 submetacentric chromosome (Carey and Mather 1999).

### Life history variation in *L. calcarifer* and links to chromosomal inversions

BP are considered a catadromous species, which exhibit protandrous hermaphroditism. There are indications that both sexual system and life history exhibit variation across the species range. Much of the understanding of BP life history variation is based on research in Australia leaving much of the range of this taxon understudied. Therefore, there exists a potential bias in the characterization of life history variation in this lineage. Examination of the BP sexual system documented in the literature indicates that BP exhibit primary females, may not change sex from male to female, and—uncommonly—exhibit synchronous hermaphrodism (e.g. Moore 1980; Davis 1982; Garrett 1987). The transition from male to female may occur at varied times and sizes and is related to individual growth rate (Roberts *et al*. 2021), and some protandrous individuals may transition to females before spawning (Crook *et al*. 2017). Reports on sexual transition from male to female are lacking from Asian BP (Grey 1987), and BP from Songkhla Lake in Thailand appear to be gonochoristic (with separate sexes; Davis 1987). Thailand in particular is a region of high genetic diversity with all 3 karyotypes of all of the candidate inversions being present (e.g.Yue *et al*. 2009; Campbell and Becker under review; Fig. 5). Intensive fishing pressure has been hypothesized as 1 force leading to younger and smaller transitions from male to female in Asian BP compared with Australian BP, making a sexual transition hard to detect (Grey 1987). Recent sources describing and quantifying the sexual system and life history of IND and SEA lineage BP were not found in the literature (e.g. Parazo *et al*. 1998), making it difficult to make inferences regarding variation in sexual system throughout the range of BP. Certainly, large-scale inversions could be 1 mechanism that promotes diversity of sexual systems in a wide-ranging species. However, it is important to note that no GO term with obvious links to sexual development or sex determination was enriched in protein-coding genes within the candidate inversions.

Sex determination and other life history factors in BP are likely a combination of heritable (genetic and epigenetic) factors and environmental influences. The large amount of genetic variation within the 3 candidate inversions may contribute to alternative sexual systems or timing of sexual transition in BP. For aquaculture systems, predictable and controllable sex would provide enormous benefits. Further evaluation of the phenotypic role, the candidate inversions through linking homokaryotypes may be useful in understanding sex determination and sexual development in BP.

Life history development is another phenotype that varies between different populations of BP. Many populations show facultative catadromy and partial migratory behavior—i.e. when not all individuals within a population migrate to freshwater (Crook *et al*. 2017; Roberts *et al*. 2024). Given what is known about migratory behavior from other species of fish (e.g. Salmonidae), it is likely that life history development has a substantial environmental component and may be influenced by, for example, monsoonal strength (Roberts *et al*. 2024). Recent species descriptions of *Lates lakdiva* from Sri Lanka and *Lates uwisara* from Myanmar have highlighted phenotypic diversity in the *L. calcarifer* sensu *lato* group, with the description of *L. uwisara* noting its large size (Pethiyagoda and Gill 2012). In this light, broader scale linking of phenotype to genotype with Genome-Wide Association Study (GWAS), such as combining otolith microchemistry (to quantify migratory behavior) or sexual system data with genomic-scale epigenomic or sequence data, may provide a link between phenotype and heritable variation (e.g. Campbell *et al*. 2022). Associated variants with different life history strategies may have various relationships to phenotypes of interest, such as spatially variable selection or genotype-dependent habitat choice (Pavey *et al*. 2015). In the study of the rainbow trout Omy05 inversion, initial above-barrier populations were noted to have increased frequency of the inverted forms (e.g. Pearse *et al*. 2014, 2019), leading to the hypothesis that life history development was due, in part, to the Omy05 karyotype. However, follow-up studies report the association of the Omy05 inversion depends on latitude, and it appears to be less crucial in life history development in high-latitude populations (Pearse *et al*. 2019; Weinstein *et al*. 2019; but see Arostegui *et al*. 2019). GO enrichment analyses did find examples of terms with possible links to life history development—such as response to cation stress and response to osmotic stress—associated with protein-coding genes found within candidate inversions. However, the functions associated with the enriched GO terms are many and varied and making conclusions regarding selective pressure maintaining different forms of the candidate inversions is not advised without GWAS-based approaches, such as those mentioned above.

### Roles of chromosomal inversions

The 3 main lineages of BP may be viewed as separate or incipient species with a potential natural contact zone between the IND and SEA lineages. This leads to the key question of how do species persist and form in the face of gene flow? Definition of what the separate lineages are relies on systematic ichthyological work beyond the scope of the current study. However, chromosomal inversions have a strong role in answering fundamental evolutionary questions regarding speciation as inversions reduce gene flow either through promoting sterility or through suppressing recombination and keeping linked adaptive variants intact (Noor *et al*. 2001; Rieseberg 2001). For example, lacustrine speciation in darters (*Etheostoma*: Percidae) where there are no barriers to gene flow involves an inversion with homology to Omy05 as well as homology to an Atlantic Cod inversion associated with local adaptation (MacGuigan *et al*. 2023). Key genes of *clocka*, *prdx6*, *nrl*, and *kita* implicated in the study of MacGuigan *et al*. (2023) are found on Lca017 in the BP genome annotations, and Lca017 is shown by macrosyntenic comparison to have homology to Omy05 (Fig. 7). However, we found no evidence that syntenic regions of the BP genome to Omy05 or the less well-characterized Omy20 (Campbell *et al*. 2021; Hale *et al*. 2024) were part of any candidate inversion.

A series of outstanding question regarding genetics in BP remain. For example, is the hybridization apparent between the IND and the SEA lineages due to human-mediated translocation and subsequent release? Is hybridization occurring during artificial propagation? Or is it natural hybrid zone? BP are large and able swimmers capable of dispersal leading to contact between major BP lineages. This would lend support to a natural hybrid zone hypothesis. However, BP genetic structure in Australia so closely reflects freshwater systems, that—at least in part of its range—it may be considered a freshwater fish from a genetic structure viewpoint making natural introgression unlikely (e.g. Marshall 2005). That being said, the IND and SEA BP may have a greater marine occupancy and dispersal tendency leading to increased gene flow in SEA and contact between the lineages compared with AUS + NG BP. Inversions could be under selection if they act to maintain co-adapted genes and alleles, and individuals within SEA exhibit various inversion genotypes from the 3 inversion chromosomes (Supplementary Fig. 5), suggesting selection, in some capacity, is maintaining their variation. As mentioned above, linking adaptive phenotypes to different inversion karyotypes would be a promising area of future research in understanding how and why selection has maintained this variation.

### Conclusion

BP are a widespread species complex with diverse selective pressures and evidence of phenotypic variation across their range. Using population genetic approaches that aim to locate regions of the genome that show patterns of population segregation that are different from the majority of the genome, we identify 3 candidate chromosome inversions in BP. Subsequently, we characterize the distribution of these inversions across phylogenetic and geographic scales. The roles of these major structural variants in growth, life history variation, sexual system, fitness, and maintenance of species boundaries all remain to be explored and may provide exceptional benefits to the production and management of this highly valuable species.

## Supplementary Material

jkae141_Supplementary_Data

## Data Availability

All raw sequence data analyzed in this study are available from the National Center for Biotechnology Information (NCBI) under BioProject accessions PRJNA311498, PRJNA1021005, and PRJDB3890. Candidate inversion genotypes for each individual as well as sample metadata are provided in Supplementary Table 1. Codes for the generation of figures and intermediate files are available at https://github.com/MacCampbell/g3-lates-inversions and https://doi.org/10.5281/zenodo.12176402. Supplemental material available at G3 online.
