# Supplementary material for: Genomic structural variation in Barramundi Perch Lates calcarifer and potential roles in speciation and adaptation
Source: G3 (Bethesda). 2024 Jun 27;14(8):jkae141. doi: 10.1093/g3journal/jkae141 (PMC11817853; doi:10.1093/g3journal/jkae141)
Supplement: jkae141_Supplementary_Data [file jkae141_supplementary_data.zip › Supplemental_Figure_Legends_G3-2024-405192.docx]

**Supplemental Figure S1** Individual heterozygosity boxplots of combined RAD and WGS data sets from 16,306 SNPs presented by major lineage. Australia + New Guinea (AUS+NG), Southeast Asia (SEA) and Indian Subcontinent (IND). The plots are presented for 130 RAD sequenced individual (AUS+NG n=57, SEA n = 76) and 60 WGS individuals (AUS+NG n = 17, IND n = 13, SEA n = 30) in separate facets.

**Supplemental Figure S2** Multidimensional Scaling (MDS) outputs from local Principal Component (PC) analysis. The first three MDS axes are displayed with outlier points indicated in black.

**Supplemental** F**igure S3** Linkage Disequilibrium (LD) analysis of RAD data set (76,601 called SNPS) of three chromosomes exhibiting increased LD (A = Lca03, B = Lca05, C = Lca20) and a fourth chromosome without increased LD for comparison (D = Lca01). The *x* – axis is the position of a SNP, ‘SNP A’, with the *y* – axis the position of a second SNP, ‘SNP B’. Each point is color coded to the measurement of LD (R2).

**Supplemental Figure S4** Principal Component (PC) analyses of the three candidate chromosomal inversion regions split between RAD data type (n = 130) and WGS data type (n=60). Main lineage is indicated by shape and collection locality by color as in legend. Panel A from Lca03 is based on the analysis of 1,983 SNPs, panel B Lca05 on 2,517 and panel C Lca20 on 2,494.

**Supplemental Figure S5** Candidate inversion genotypes across main Barramundi Perch lineages of Australia + New Guinea (AUS+NG, n = 71 individuals), Southeast Asia (SEA, n = 106 individuals) and the Indian subcontinent (IND, n = 13 individuals) at an individual level.
