# Supplementary figures and images for: Genomic structural variation in Barramundi Perch Lates calcarifer and potential roles in speciation and adaptation
Source: G3 (Bethesda). 2024 Jun 27;14(8):jkae141. doi: 10.1093/g3journal/jkae141 (PMC11817853; doi:10.1093/g3journal/jkae141)

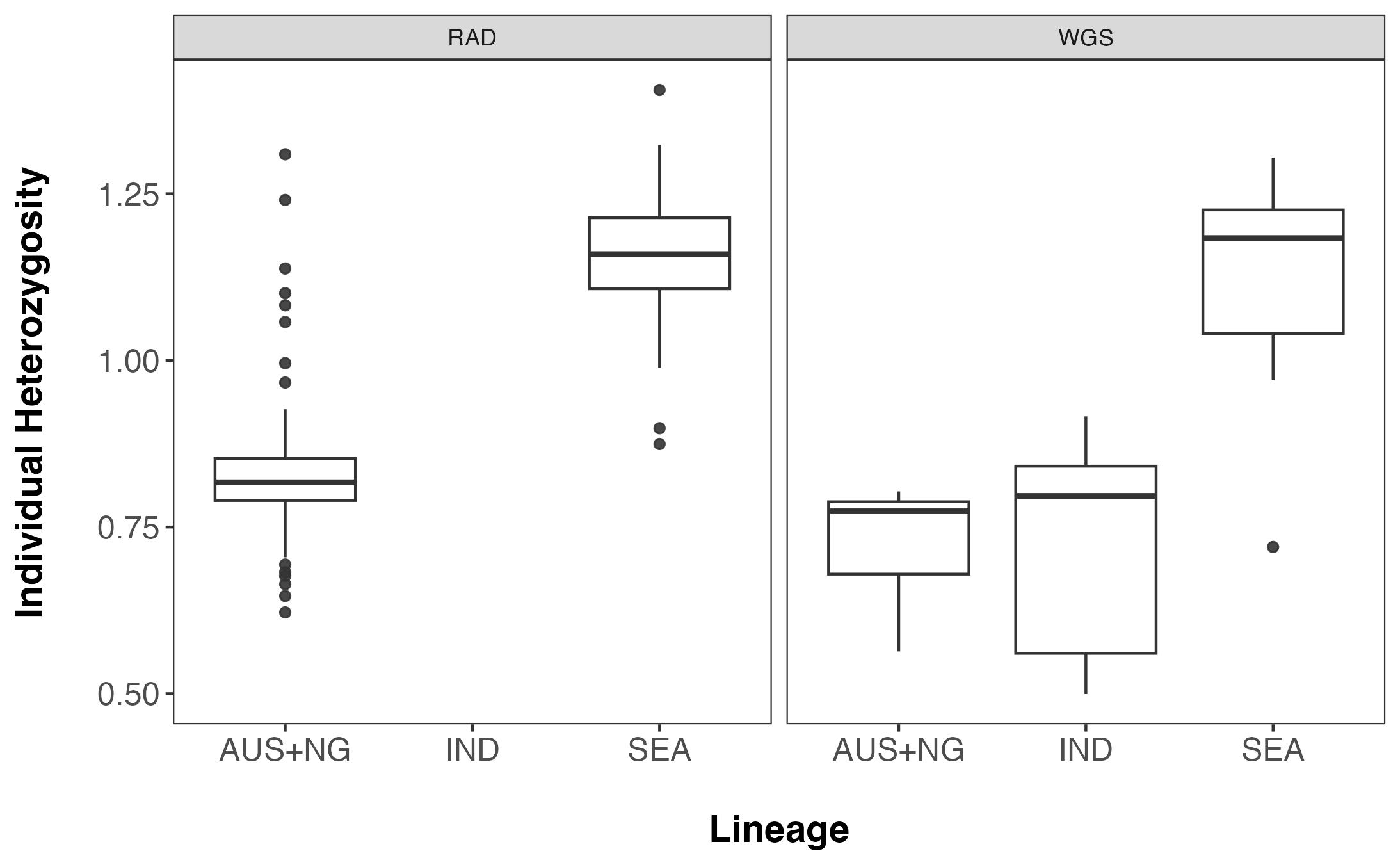

Supplement: jkae141_Supplementary_Data [file jkae141_supplementary_data.zip › Supplemental_Figure_S1_G3-2024-405192.jpg]

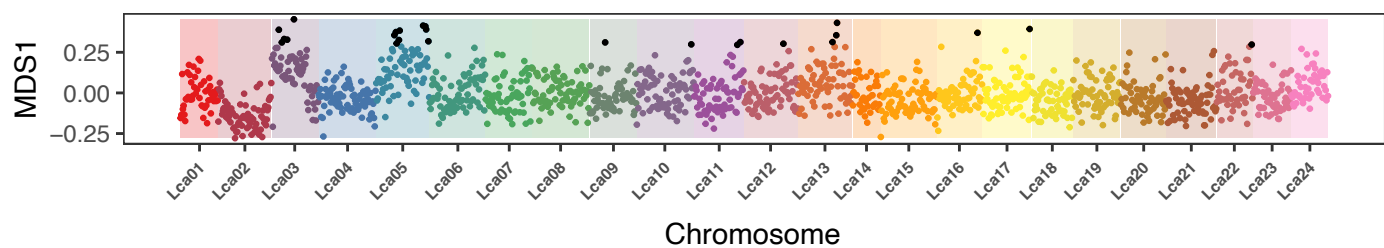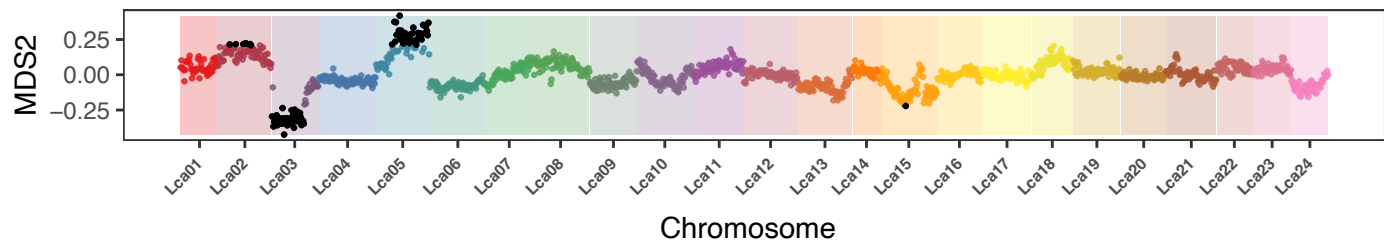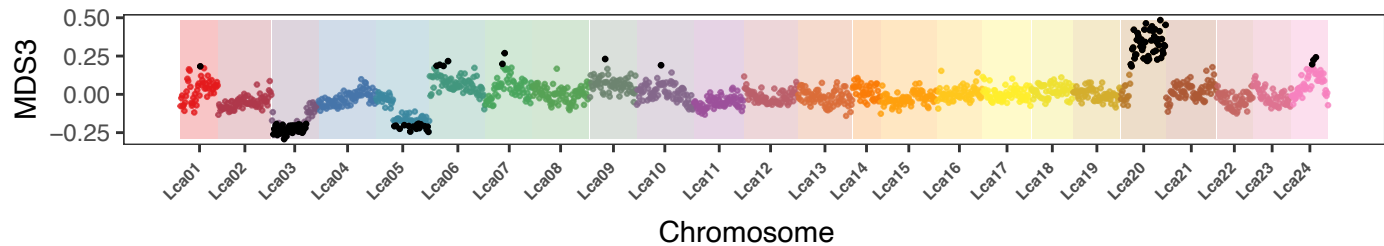

Supplement: jkae141_Supplementary_Data [file jkae141_supplementary_data.zip › Supplemental_Figure_S2_G3-2024-405192.pdf]

**A. Lca03**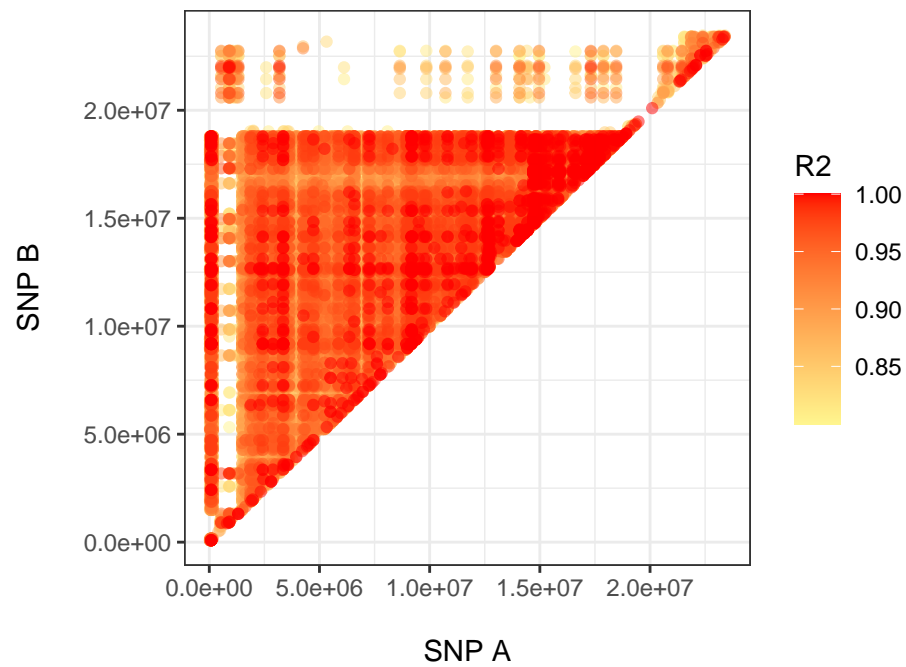**B. Lca05**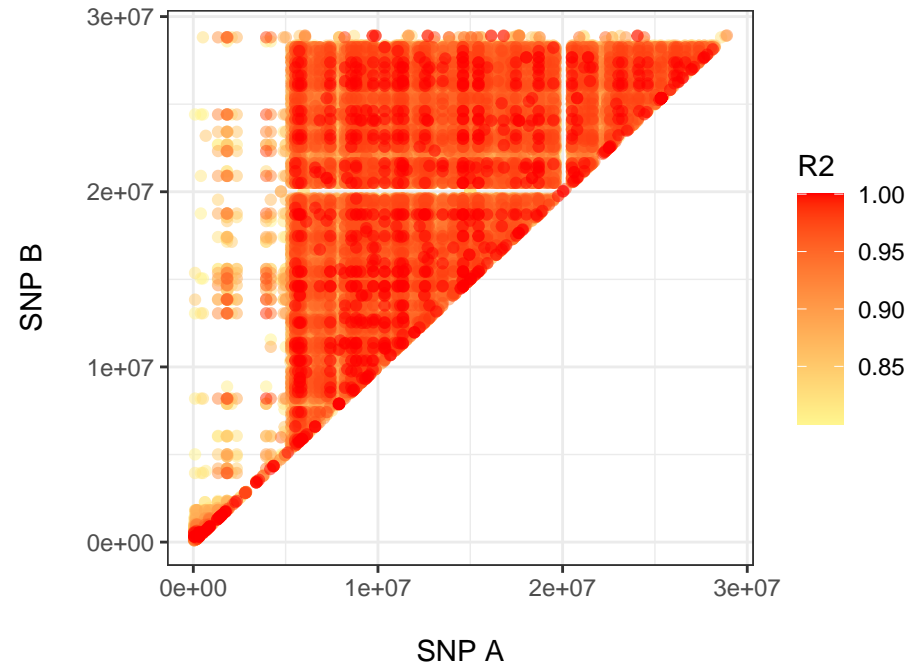**C. Lca20**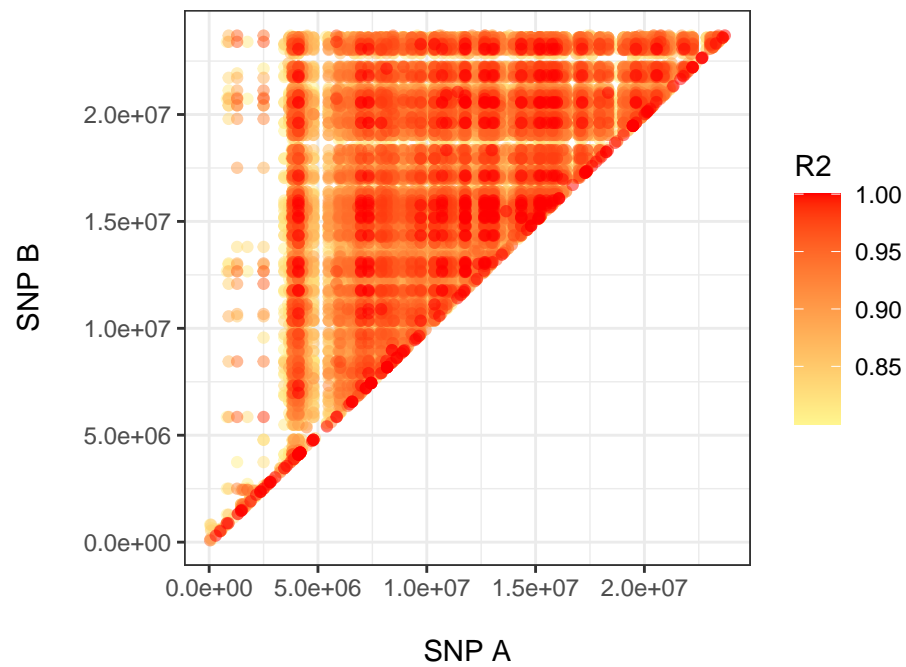**D. Lca01**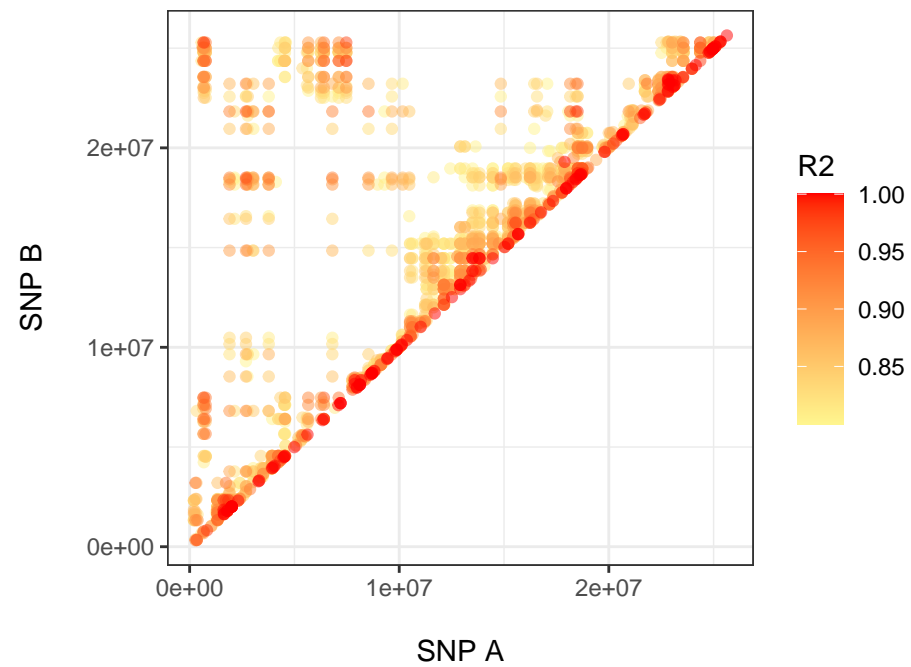

Supplement: jkae141_Supplementary_Data [file jkae141_supplementary_data.zip › Supplemental_Figure_S3_G3-2024-405192.pdf]

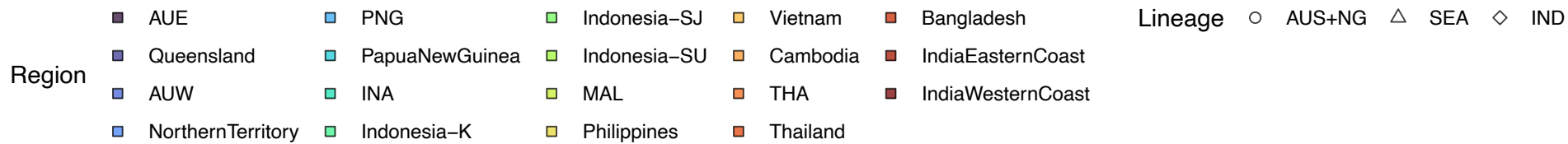

## A. Lca03

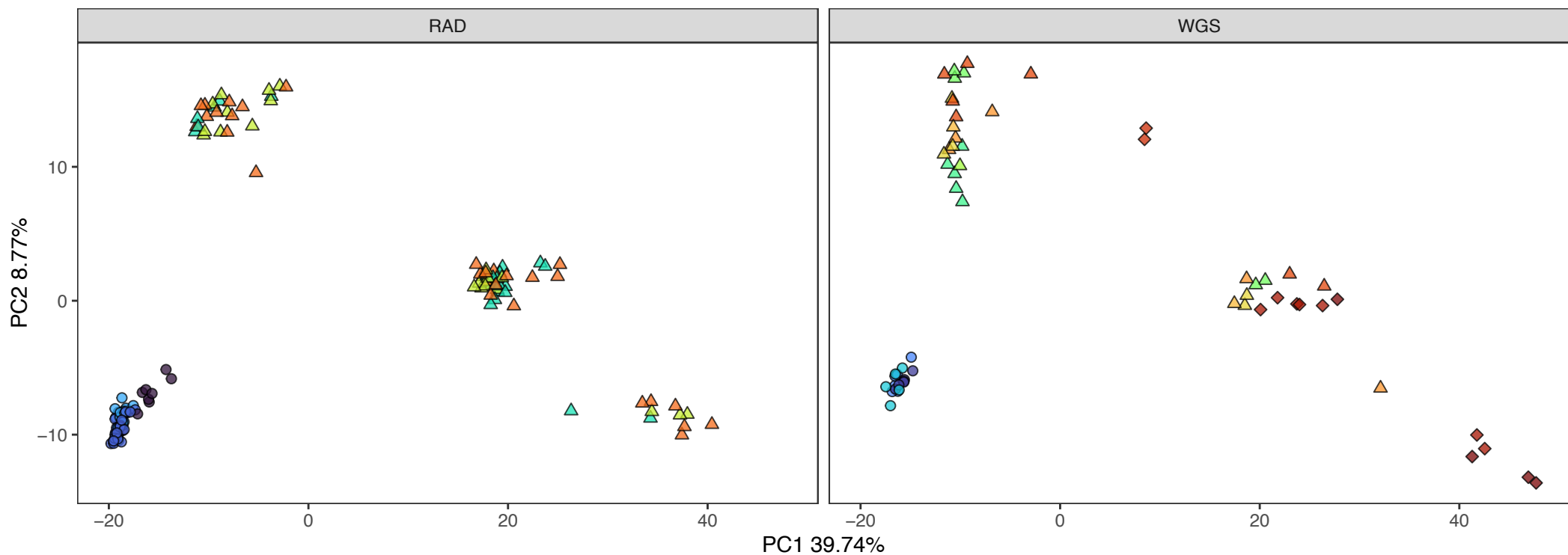

## B. Lca05

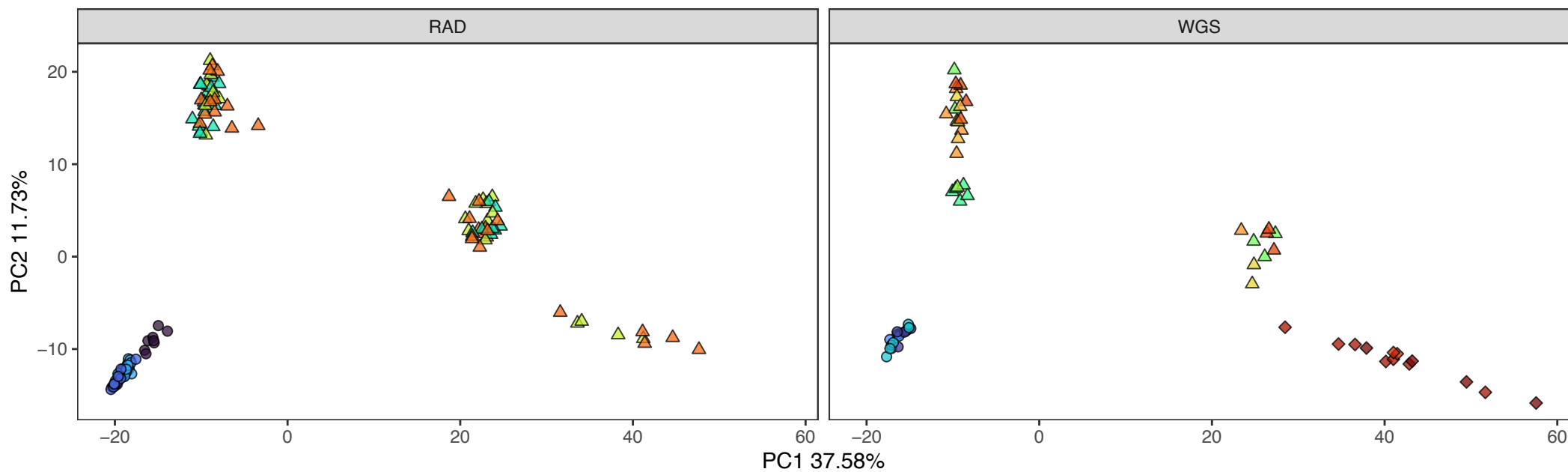

## B. Lca20

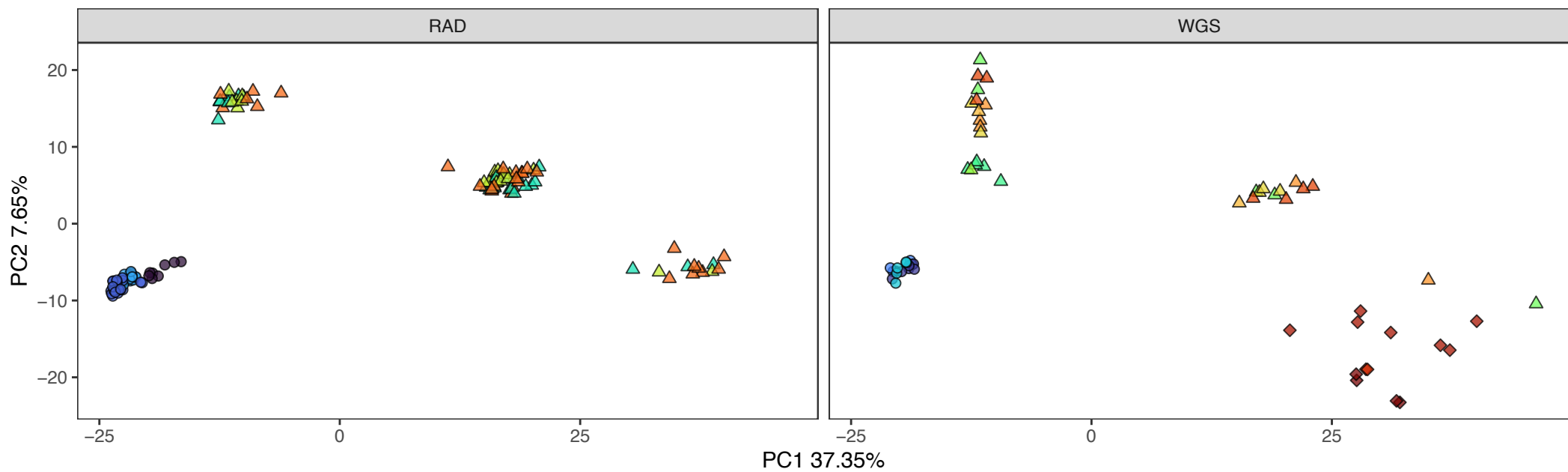

Supplement: jkae141_Supplementary_Data [file jkae141_supplementary_data.zip › Supplemental_Figure_S4_G3-2024-405192.pdf]

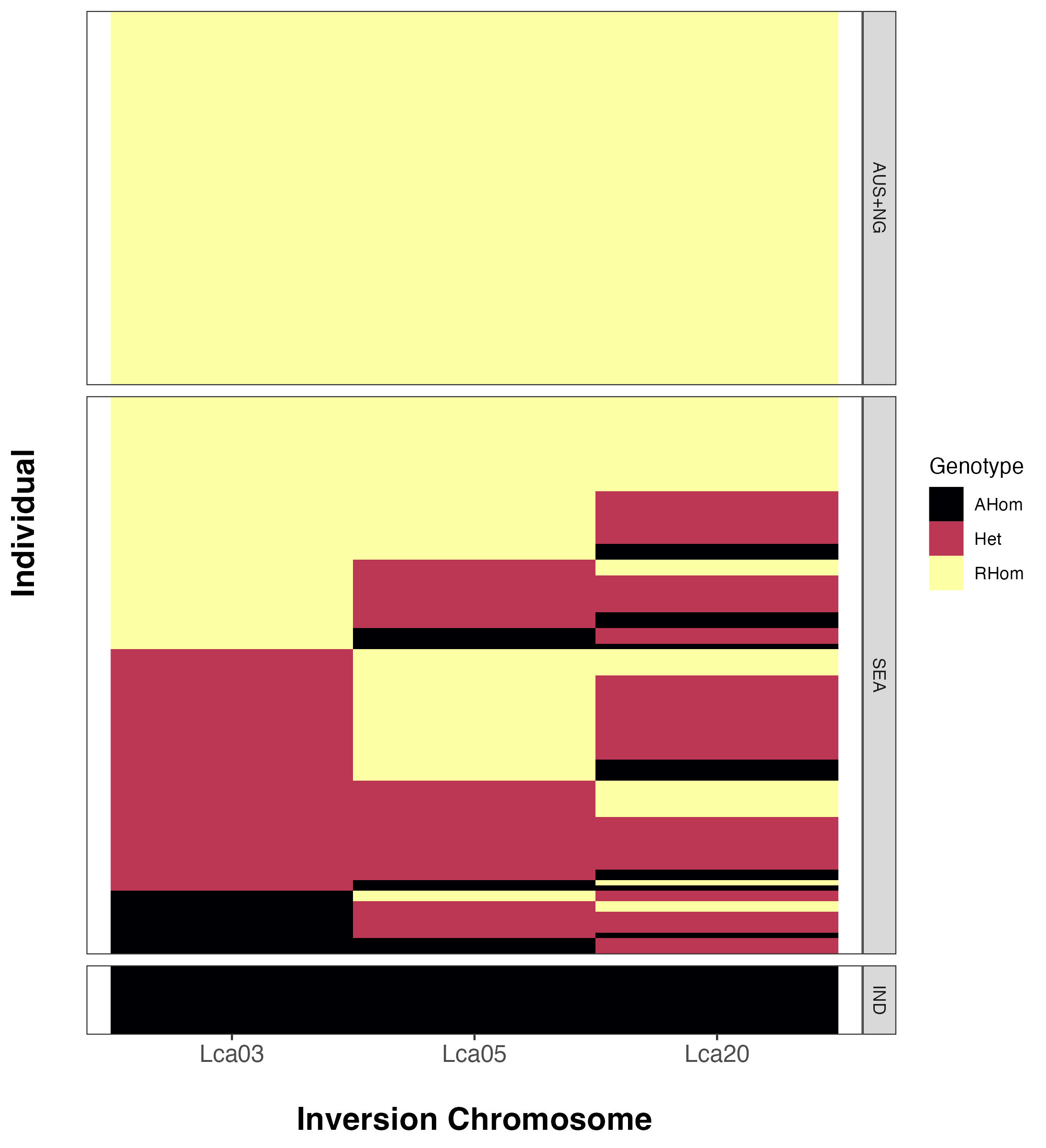

Supplement: jkae141_Supplementary_Data [file jkae141_supplementary_data.zip › Supplemental_Figure_S5_G3-2024-405192.jpg]
